# TMEM225 Is Essential for Sperm Maturation and Male Fertility by Modifying Protein Distribution of Sperm in Mice

**DOI:** 10.1016/j.mcpro.2024.100720

**Published:** 2024-01-20

**Authors:** Zheng Lv, Longjie Sun, Xiaomei Xie, Xiaohong Yao, Shuang Tian, Chaofan Wang, Fengchao Wang, Jiali Liu

**Affiliations:** 1 State Key Laboratory of Animal Biotech Breeding, College of Biological Sciences, China Agricultural University, Beijing, China; 2 Transgenic Animal Center, National Institute of Biological Sciences, Beijing, China; 3 Tsinghua Institute of Multidisciplinary Biomedical Research, Tsinghua University, Beijing, China

**Keywords:** infertility, transmembrane protein 225, sperm maturation, asthenospermia, ROS

## Abstract

Nonobstructive azoospermia is the leading cause of male infertility. Abnormal levels of transmembrane protein 225 (TMEM225), a testis-specific protein, have been found in patients with nonobstructive azoospermia, suggesting that TMEM225 plays an essential role in male fertility. Here, we generated a *Tmem225* KO mouse model to explore the function and mechanism of TMEM225 in male reproduction. Male *Tmem225* KO mice were infertile. Surprisingly, *Tmem225* deletion did not affect spermatogenesis, but TMEM225-null sperm exhibited abnormalities during epididymal maturation, resulting in reduced sperm motility and an abnormal hairpin-loop configuration. Furthermore, proteomics analyses of cauda sperm revealed that signaling pathways related to mitochondrial function, the glycolytic pathway, and sperm flagellar morphology were abnormal in *Tmem*225 KO sperm, and spermatozoa lacking TMEM225 exhibited high reactive oxygen species levels, reduced motility, and flagellar folding, leading to typical asthenospermia. These findings suggest that testicular TMEM225 may control the sperm maturation process by regulating the expression of proteins related to mitochondrial function, glycolysis, and sperm flagellar morphology in epididymal spermatozoa.

## Highlights

• TMEM225-null mice develop asthenospermia and are infertile.
• TMEM225 is essential for sperm maturation in mouse epididymis.
• TMEM225-null sperm showed high ROS levels, reduced motility, and flagellar folding.

## Graphical Abstract

**Figure:**
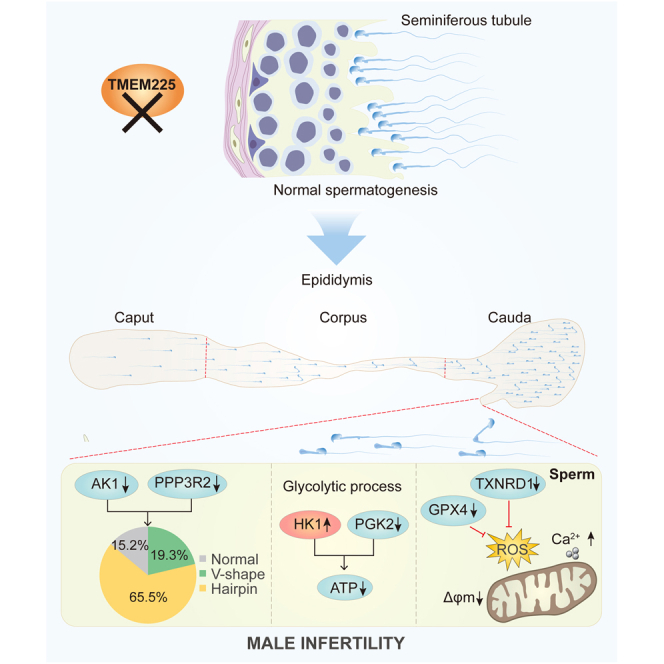

## In Brief

Previous studies have found abnormal transmembrane protein 225 (TMEM225) expression levels in patients with nonobstructive azoospermia. Here, we constructed a *Tmem225* knockout mouse model and analyzed the function and mechanism of TMC7 in male reproduction using Label free. Our results suggest that TMEM225 regulates sperm maturation by affecting mitochondrial function, glycolysis, and sperm flagellar morphology related proteins.

Approximately 8% to 12% of couples worldwide are affected by infertility, and approximately 50% of infertility cases are caused by male factors (1). Thus, healthy sperm are essential for oocyte fertilization and the development of a fertilized egg (2). Sperm integrity, sperm organelles, sperm motility, sperm functionality, and sperm proteins are indicators of healthy sperm (3). Spermatogenesis, in which diploid germ cells develop into spermatozoa, occurs in the testis (4). At this time, the spermatozoa exhibit sperm integrity and a well-arranged organelle structure but cannot move or fertilize. The sperm are expelled from the seminiferous tubule, processed by the epididymis, and stored in the cauda epididymis; the maturation process is completed, and all indicators of healthy sperm should be present (5). Spermatids are highly specialized cells that are transcriptionally silent and therefore change only through the acquisition or loss of proteins (6, 7, 8). Several previous studies on sperm proteomics in the epididymis have shown that sperm maturation is closely related to protein changes (9, 10, 11). The molecular mechanisms of spermiogenesis and sperm maturation have not been fully elucidated.

The membrane proteins in sperm are involved in spermiogenesis and sperm maturation. ARMC12, a mitochondrial peripheral membrane protein, regulates the temporal and spatial dynamics of mitochondria and forms the mitochondrial sheath through synergistic interactions with multiple proteins on the surface of sperm mitochondria (12). ADAM24, a plasma-anchored sperm protease, is involved in sperm function during maturation or fertilization (13). Testis expressed gene 101 (TEX101) does not affect sperm maturation but is essential for spermatogenesis; moreover, this molecule has been used to clinically evaluate success after vasectomy and azoospermia (14, 15, 16). In humans, the membrane bound O-acyltransferase domain containing 1 (MBOAT1) mutation is associated with male infertility (17). Loss of cilia and flagella associated protein 65 (CFAP65), a transmembrane protein, led to severe asthenospermia and defects in the sperm head and cilium in male mice (18).

Previous research revealed abnormal protein levels of transmembrane protein 225 (TMEM225) in infertile male patients with nonobstructive azoospermia (19). This protein contains four transmembrane domains and is expressed solely in the rat testis (20). Researchers subsequently found that TMEM225 is expressed in germ cells only after the elongated spermatozoa stage in mice (21). However, the physiological importance and molecular mechanism of spermiogenesis remain unclear. Thus, by constructing a KO mouse model, we discovered the crucial function of TMEM225 in mouse sperm maturation.

## Experimental Procedures

### Animals

*Tmem225*^−/−^ mice were generated using the CRISPR/Cas9 system. *Tmem225* KO mouse guide RNAs were designed using the CRISPR guide-design web tool (https://crispr.dbcls.jp/). The selected paired gRNA sequences were ggtggtcaggcggataatgAGG and CCAagcacgtcgggtaacctggg. Mouse zygotes obtained by mating males with superovulated C57BL/6J females were injected with a mixture of Cas 9 mRNA (80 ng/μl), single gRNA1 (40 ng/μl), and single gRNA2 (40 ng/μl). Injected zygotes were transferred into pseudopregnant CD1 female mice, and viable adult mice were obtained. The sequences of the primers used for genotyping are shown in Supplemental Table S1. All the experiments were conducted according to the established guidelines and with the approval of the Institutional Animal Care and Use Committee of China Agricultural University (No. AW80401202-3-7). All mice in this study were of the C57BL/6 strain. All mice were bred and housed under specific pathogen-free conditions at a controlled temperature (22 ± 1 °C) and exposed to a constant 12-h light–dark cycle in the animal facilities of China Agricultural University.

### TMEM225 Antibody Generation

The TMEM225 rabbit antibody was generated according to previous methods (21). Briefly, rabbit polyclonal anti-TMEM225 antibodies were raised against a keyhole limpet hemocyanin-conjugated peptide corresponding to the carboxy-terminal region of mouse TMEM225 (217NRPHTQARRVTWAL230).

### Western Blotting

The protein lysates (40 μg total protein/lane) were electrophoretically separated with sodium dodecyl sulfate and polyacrylamide gels and electrically transferred to polyvinylidene fluoride membranes (IPVH00010, Millipore). The membranes were blocked in 5% skim milk for 1 h and incubated with the primary antibody (Supplemental Table S2) overnight at 4 °C. Then, the membranes were incubated with secondary antibodies (Supplemental Table S2) for 2 h. The proteins were visualized using a Tanon 5200 chemiluminescence imaging system and a SignalUp Western detect sensitizer (P0272, Beyotime).

### Sample of the Epididymal Epithelium

The cauda epididymis of adult WT mice was added to PBS and incubated for 10 min. After the sample was mixed and inverted, the cauda epididymis was left for 1 min to settle naturally, and the supernatant was discarded. The procedure was repeated three times to obtain epididymal epithelial tissue without sperm.

### Fertility Test

The tested male mice were fed in the same area as the WT female mice for 6 months; during this period, the litters born to the female mice were counted.

### Mating Test

Each male mouse to be tested was fed separately; two WT female mice were placed in the cage with the male at 5 PM every day, and the female mice were observed for vaginal plugs at 8 AM the next day. This procedure was repeated for 15 days, after which the number of females with vaginal plugs was counted.

### Immunofluorescence and histological Analysis

The mouse tissue was fixed in 4% paraformaldehyde (P6148-500G, Sigma-Aldrich) with 0.1 M PBS, dehydrated with graded ethanol solutions, vitrified with xylene, and embedded in paraffin. The sections, which were 5 microns thick, were expanded in distilled water at 42 °C for 1 min, adhered to the slides, and allowed to dry. The tissue sections were rehydrated with xylene and graded ethanol solutions. Histological analysis of the testes was performed using periodic acid–Schiff staining, and histological analysis of the epididymides was performed using H&E staining. Thereafter, a Ventana DP 200 digital pathological biopsy scanner was used for imaging. For the immunofluorescence assay, sections were microwaved for antigen retrieval in sodium citrate buffer (pH 6.0) after rehydration. Nonspecific antigens were blocked with 10% goat serum (prepared with 0.1 M PBS) at room temperature for 1 h. The sections were incubated with a 5% goat serum-diluted primary antibody (Supplemental Table S2) overnight at 4 °C. Then, the sections were incubated with the secondary antibody (Supplemental Table S2) at room temperature in the dark for 2 h. The slides were sealed with a fluorescent quenching agent containing 4,6-diamidino-2-phenylindole (P0131, Beyotime). Photographs were taken using a Nikon A1 laser scanning confocal microscope.

### Sperm Count

The cauda epididymis was added to PBS and incubated at 37 °C for 15 min to allow the sperm to fully swim out. The sperm count in each cauda epididymis was calculated using a blood counting chamber.

### Analysis of Sperm Motility

Human tubal fluid (HTF) solution (MR-070, Sigma-Aldrich) was pre-equilibrated for 2 h in a constant temperature incubator at 37 °C. The cauda epididymis was separated and placed into 500 μl of HTF. After 15 min of swim-out in an incubator at 37 °C, sperm motility in the supernatant was analyzed using a sperm analysis system (CASA system version12, CEROS, Hamilton Thorne Research). More than 400 sperm were counted for each sample.

### Sperm Morphology Assay

Epididymal sperm were released in PBS, and the appropriate solution was evenly coated on clean adhesive slides. The sperm were treated with 4% paraformaldehyde (prepared with 0.1 M PBS) for 10 min and then dried at room temperature to generate sperm smears. The sperm smears were washed with 0.1 M PBS three times for 5 min. Subsequently, the sperm were dyed with 0.22% Coomassie brilliant blue stain (Coomassie Brilliant Blue G-250, Thermo Fisher Scientific) (containing 50% methanol, 10% glacial acetic acid, and 40% water) for 2 min, washed with water and sealed with 50% glycerin. Imaging was performed using a Ventana DP 200 digital pathological biopsy scanner.

### Sperm Immunofluorescence

Epididymal sperm smears (from the Sperm Morphology Assay section) were treated with 0.5% Triton X-100 (T9284, Sigma-Aldrich) for 10 min. The blocking of nonspecific antigens and binding of antibodies followed the same procedure used for immunofluorescence and histological analysis. Images were taken using a Nikon fluorescence microscope.

### Acrosome Reaction Assay

The cauda epididymis was added to the HTF to obtain a sperm suspension. The sperm were diluted to 5 × 10^6^ per milliliter after 15 min at 37 °C. The sperm suspension was incubated at 37 °C for 2 h to capacitate the sperm. Then, progesterone (P0130, Sigma-Aldrich) was added to the suspension at a final concentration of 150 μM. The mixture was cultured at 37 °C for 15 min to induce an acrosome reaction. Finally, sperm smears were obtained, and the acrosome reaction was analyzed using Coomassie blue staining.

### Determination of Relative Calcium (Ca^2+^) Levels

The relative calcium level of sperm in the cauda epididymis was determined using a Fluo-4 AM calcium ion concentration detection kit (CA1190, Solarbio). An equal amount of 20% Pluronic F127 was added to a Fluo-4 AM (5 mM) solution and diluted with Hanks' Balanced Salt Solution (HBSS) to 5 μΜ. Sperm in the cauda epididymis were released in HBSS. Then, the sperm suspension was diluted to 10^5^ sperm/ml. Sperm were collected by centrifugation at 1200 rpm for 5 min and then cultured at 37 °C for 20 min with the prepared working solution for calcium ion detection. Five volumes of HBSS containing 1% fetal bovine serum were added, and the culture was continued for 40 min. The sperm were washed and suspended three times in Hepes-buffered saline and cultured at 30 °C for 10 min. Nikon fluorescence microscopy was used to observe and photograph each sperm, and ImageJ (https://imagej.net/) was used to analyze the relative levels of calcium ions in each sperm.

### Transmission Electron Microscopy (TEM)

Fresh cauda epididymis tissue was removed and fixed overnight at 4 °C with 2.5% glutaraldehyde and 1% paraformaldehyde (diluted in PBS). The tissue was cut into 1-mm pieces and washed three times with 0.1 M PBS for 10 min each. The tissue was dehydrated with gradient ethanol and treated with pure propylene oxide for 15 min. Then, 25% Spurr’s resin, 50% Spurr’s resin, and 75% Spurr’s resin (prepared with propylene oxide) were used to replace the solution and incubated for 4 h each. Pure Spurr’s resin was used for replacement and incubated for more than 8 h before polymerization at 70 °C for 14 h. A Leica EM UC7 ultrathin microtome was used to cut the ultrathin sections at an 80 nm thickness using a DiATOME diamond knife. The ultrathin sections were cut on a single-hole carrier net covered with a Formvar membrane. The ultrathin sections were stained with 2% uranium dioxyacetate for 15 min and lead citrate for 10 min. Images were taken using a Hitachi HT7800 TEM.

### Measurement of Sperm Reactive Oxygen Species

The relative ROS level in the sperm was measured using a ROS assay kit (S0033, Beyotime). After the sperm swam out from the cauda epididymis, they were centrifuged at 1200 rpm for 5 min and collected in a centrifuge tube. A 10 μM 2′,7′-diacetyldichlorofluorescein-diacetate (diluted in PBS) solution was added, and the mixture was incubated at 37 °C for 20 min. Nikon fluorescence microscopy was used to observe and photograph each sperm, and ImageJ was used to analyze the fluorescence intensity of each sperm (Supplemental Fig. S6, *A* and *B*).

### Sperm JC-1 Assay

The membrane potential of the sperm mitochondria in the cauda epididymis was determined using JC-1 (C2005, Beyotime). After the sperm were obtained from the cauda epididymis, a JC-1 working solution (5 μg/ml) was prepared with PBS to achieve a final sperm concentration of 10^6^ sperm/ml. After incubation at 37 °C for 20 min, the sperm were washed three times with PBS and then suspended. Nikon fluorescence microscopy was used to observe and photograph each sperm, and ImageJ was used to analyze the fluorescence intensity of each sperm.

### Sperm ATP Assay

The sperm of the cauda epididymis were released in 1 ml of PBS, and the upper fluid was transferred to a new centrifuge tube. The sperm count was calculated by taking 10 μl of the remaining liquid and centrifuging it at 1200 rpm for 5 min. The ATP level in the sperm was determined according to the protocol of the ATP assay kit (S0026, Beyotime). The fluorescence intensity was analyzed using the Tecan Spark multifunctional enzyme label instrument. The calculated relative ATP level was corrected using the sperm count (Supplemental Fig. S5, *A* and *B*).

### Label-free Quantitative Proteome Assay

The cauda epididymis of each mouse was cut and placed in PBS, cut in half with ophthalmic scissors and incubated at 37 °C for 15 min to allow the sperm to fully swim out. The supernatant was centrifuged at 3000 rpm for 3 min, the upper liquid was removed, and the amount of precipitate was measured. Lysis buffer (2 M thiourea, 7 M urea, 1% CHAPS) was added to the sample, which was subsequently mixed by swirling. The sample was incubated at 30 min and centrifuged at 13,000 rpm (15,700*g*) and 4 °C for 15 min. The supernatant was collected, and the protein concentration was determined by the Bradford method. Twenty-five micrograms of protein was extracted from each sample and subjected to enzymatic digestion into peptides by trypsin. Liquid chromatography tandem mass spectrometry analysis was performed after Ziptid C_18_ solid-phase extraction and redissolution. The polypeptide components were dissolved in 10 μl of 0.1% formic acid (FA) and separated by EASY-nLC liquid-phase and low-pH reverse-phase C_18_ capillary chromatography (150 μm × 150 mm, 1.9 μm). Phase A consisted of 99.9% H_2_O and 0.1% FA, and phase B consisted of 80% acetonitrile, 19.9% H_2_O, and 0.1% FA. The effective elution gradient was 7 to 45%. The total elution time was 90 min, and the flow rate was 0.6 μl/min.

Gradient

**Table:** 

Time (min)	A%	B%	Flow rate (nl/min)
0	93	7	600
10:00	88	12	600
70:00	70	30	600
82:00	55	45	600
83:00	5	95	600
90:00	5	95	600

The polypeptide mixture was then analyzed and identified by an Orbitrap Q Exactive HF mass spectrometer. For the parameter settings, each full scan was a high-speed signal-dependent scan, and the scanning time was 90 min. The mass spectrometry 1 parameters were as follows: resolution of 60,000, scanning range of 350 to 1600 m/z, AGC of 3e6, and maximum injection time of 50 ms. After each mass spectrometry 1 scan, the first 40 ions were screened and smashed into the collision cell, and the collision energy was 30%. The mass spectrometry 2 parameters used were as follows: resolution of 30,000, charge state screening (containing precursors with +2 to +7 charges), and dynamic elimination for 90 s. Finally, the raw data were obtained from the mass spectrum.

### Experimental Design and Statistical Rationale

We used a label-free method to analyze proteomic changes in TMEM225-null sperm. Sperm from mice with heterozygous genotypes were selected as the control (Ctrl) group, and sperm from mice with KO genotypes were used as the KO group. Three biological replicates were used for each group to ensure data reliability. Perseus software was used to determine the correlation among the three biological replicates to quantify random errors. A group *t* test was performed with the quantitative data from the two groups of samples, and proteins with a *p* value less than 0.05 were analyzed.

### Data Analysis

The resulting mass spectrum data were retrieved by MaxQuant (version 2.0.1.0, https://www.maxquant.org/). The retrieval parameters were uniprot_mouse_88048_20220901.fasta (total number of entries in the searched database: 88048); trypsin enzyme digestion; up to 2 missed cut sites; mass errors of the parent ion and fragment ion of 10 ppm and 0.02 Da, respectively; carbamidomethyl (C) as a fixed modification; and oxidation (M) and acetyl (N-terminal) as variable modifications. Only proteins identified in at least two biological replicates with one or more unique peptides and a false discovery rate less than 1% were considered true fraction components and were used for analysis to exclude any spurious identifications.

### Statistical Analyses

We used SPSS (Version 24.0, https://spssau.com/) to analyze the data and Prism (Version 9.0, https://www.graphpad.com/) for generating the figures. The values and error bars represent the means ± SEMs. Unpaired Student’s *t*-tests were used to determine significance. The statistical significance is shown as follows: N.S.; *p* ≥ 0.05; ∗*p* < 0.05; ∗∗*p* < 0.01; ∗∗∗*p* < 0.001; and ∗∗∗∗*p* < 0.0001.

## Results

### *Tmem225* KO Mice Were Successfully Generated

**Fig. 1:**
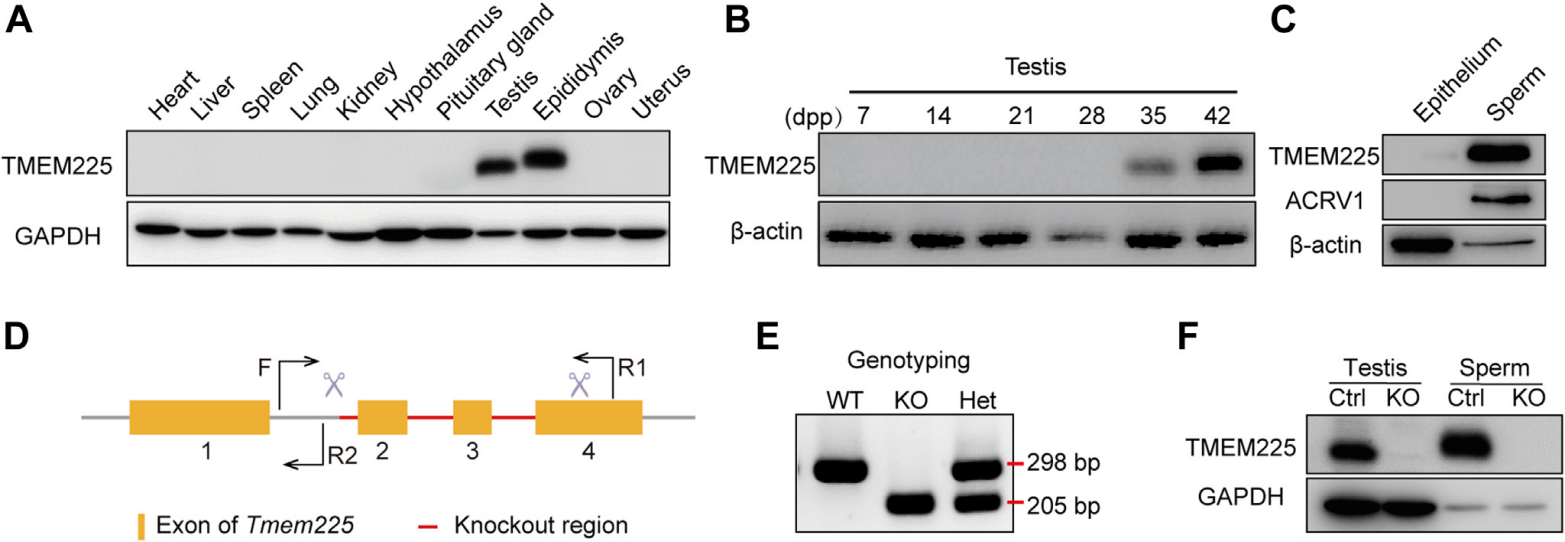
**Generation of *Tmem225* KO mice.***A*, Western blotting analysis of the TMEM225 protein in various mouse tissues. *B*, the protein expression of TMEM225 in mouse testes at different developmental stages was determined by Western blotting. *C*, expression of TMEM225 in epididymal epithelial cells and sperm. *D*, *Tmem225* KO gene strategy. F and R1 are the primers for KO mice. F and R2 are the primers for WT mice. *E*, the genotypes of the offspring of the *Tmem225* KO mice were identified by PCR. *F*, the KO efficiency of the TMEM225 protein in mouse testes and spermatozoa was analyzed *via* Western blotting. TMEM225, transmembrane protein 225.

First, we determined the TMEM225 protein expression levels in various mouse tissues. The results were consistent with those of previous research (21) and showed that TMEM225 was expressed in only the testis and epididymis (Fig. 1*A*). Western blotting was used to determine TMEM225 expression in the testes of mice at different developmental stages. The results showed that the TMEM225 protein was detectable in 35-days postpartum testes, and the protein level was increased in 42--days postpartum testes (Fig. 1*B*), suggesting that TMEM225 plays an important role in spermiogenesis. Since our results also showed that TMEM225 was expressed in the epididymis, to confirm its presence in the epididymal epithelium, we separated the epididymal epithelium and sperm for Western blotting. Sperm are indicated by the acrosomal marker acrosomal vesicle protein 1. TMEM225 was not expressed in the epididymal epithelium but was expressed in sperm (Fig. 1*C*). Therefore, to investigate the role of *Tmem225* in spermiogenesis in mice, we constructed *Tmem225* KO mice (Fig. 1*D*). Then, we used PCR to identify the mouse genotypes. The band located at 298 bp was the WT band, and the 205 bp band was the KO band. In contrast, samples from heterozygous mice showed bands at both positions (Fig. 1*E*). In subsequent experiments, mice with a heterozygous genotype were used as Ctrl mice, and mice with a *Tmem225* deletion were used as KO mice. The KO efficiency was determined by Western blotting, which confirmed that *Tmem225* was successfully knocked out (Fig. 1*F*).

### Deletion of *Tmem225* Led to Infertility in Mice

**Fig. 2:**
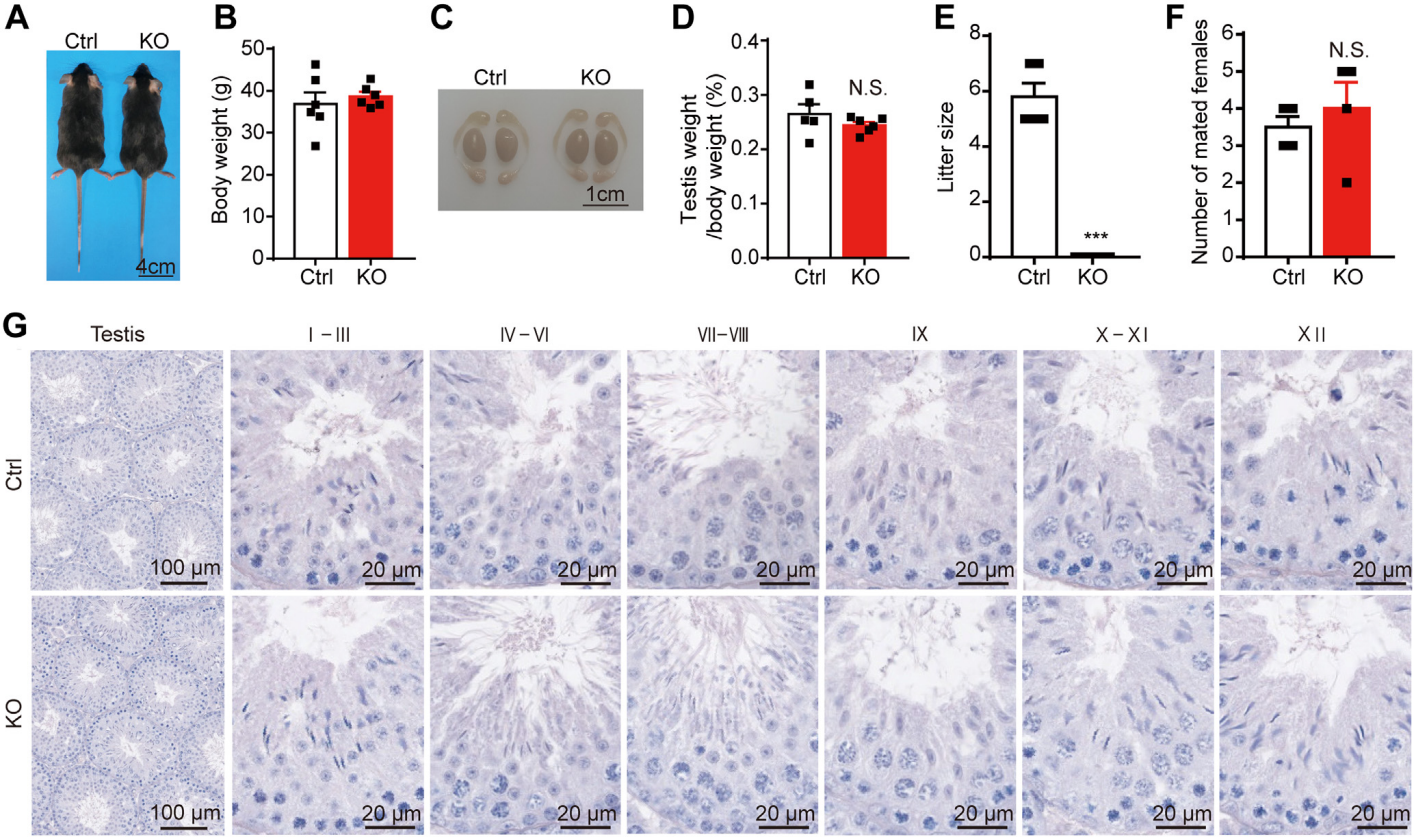
***Tmem225* KO male mice were infertile.***A* and *B*, the body sizes and body weights of 2-month-old mice were observed and analyzed. n = 3. *C*, photographs of the testis and epididymis morphology of 2-month-old mice. *D*, statistical analysis of the difference in the testis-to-body weight ratio between the Ctrl (n = 5) and KO (n = 6) mice. *E*, litter sizes of the Ctrl and *Tmem225* KO mice in the mouse fertility test. n = 5. *F*, the number of females successfully mated to the Ctrl and KO mice within 15 days n = 4. *G*, hematoxylin-eosin staining was used to observe the histology of the testis. The above data are presented as the mean ± SEM (N.S., not significantly different; ∗∗∗*p* < 0.001). TMEM225, transmembrane protein 225.

After constructing *Tmem225* KO mice, we examined the effects of this treatment on the growth and development of the KO mice. We photographed and weighed 2-month-old mice and found that *Tmem225* deletion had no significant impact on their growth or development (Fig. 2*A* and *B*). We subsequently observed that the testes and epididymides of the 2-month-old KO mice had a normal morphology (Fig. 2*C*). There was no significant difference in the testis-to-body weight ratio in the KO mice and the Ctrl mice (Fig. 2*D*). However, fertility tests revealed that the male *Tmem225* KO mice were infertile (Fig. 2*E*). We prepared mating experiments to determine whether the KO mice could mate normally. Mating experiments showed that the number of female mice with vaginal plugs after mating with the KO mice and the Ctrl mice was not significantly different (Fig. 2*F*). Based on these results, we hypothesized that spermiogenesis was impaired in the TMEM225-null mice. Consequently, we performed periodic acid–Schiff staining of paraffin sections of adult mouse testes to confirm this hypothesis. We found no significant difference in the number of seminiferous tubules at each stage between the KO and Ctrl mice (Fig. 2*G*). VASA is used as a marker of germ cells, and the expression pattern of γH2AX can distinguish different stages of spermatocytes and thus indicate the stages of seminiferous tubules. Coimmunofluorescence staining of VASA and γH2AX did not reveal significant abnormalities in spermatocytes (Supplemental Fig. S1A). Furthermore, coimmunofluorescence staining for γH2AX and the markers of late spermiogenesis hexokinase 1 (HK1) and cysteine-rich secretory protein 2 (CRISP2) revealed no significant differences between the Ctrl and KO mice (Supplemental Fig. S1, *B* and *C*). These collective data indicated that although TMEM225 is highly expressed in elongated spermatids, this molecule is dispensable for spermiogenesis.

### TMEM225 Null Mice Developed Asthenospermia

**Fig. 3:**
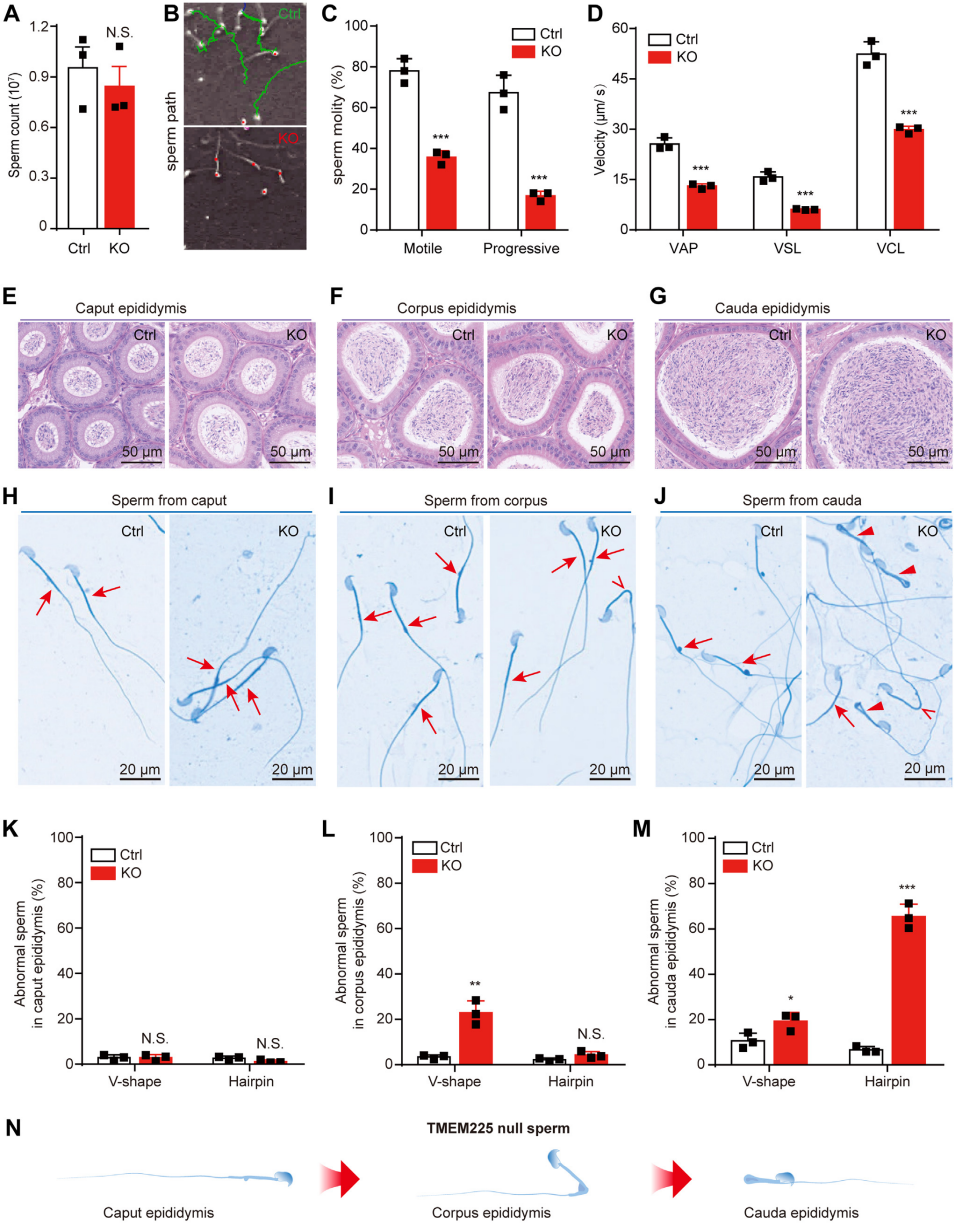
**Motility and morphological analyses of spermatozoa from TMEM225 null mice.***A*, sperm count of sperm from the cauda epididymis. n = 3. *B*, motility trajectories of spermatozoa isolated from the cauda epididymis of the control and KO mice. *Green curve*: forward movement; *red dot*: stand still. *C*, the proportion of motile and progressive spermatozoa in the cauda epididymis of the control and KO mice. n = 3. *D*, evaluation of sperm motility velocity. VAP: average path velocity, VSL: straight-line velocity, VCL: curvilinear velocity. n = 3. *E–G*, H&E staining of sections of the caput epididymis, corpus epididymis, and cauda epididymis of the Ctrl mice and TMEM225 null mice. *H–J*, coomassie brilliant blue G250 staining was used to analyze the sperm morphology of the caput epididymis, corpus epididymis, and cauda epididymis. *K*–*M*, proportions of abnormal spermatozoa in the caput epididymis, corpus epididymis, and cauda epididymis. n = 3. *N*, model diagram of morphological changes after sperm entry into the epididymis of the TMEM225 null mice. All the data are presented as the means ± SEMs (N.S., not significantly different; ∗*p* < 0.05; ∗∗*p* < 0.01; ∗∗∗*p* < 0.001). TMEM225, transmembrane protein 225.

Although spermiogenesis was not impaired in the KO mice, other processes like acrosome reaction ability, sperm motility, and sperm number are significant factors affecting fertilization (22). Therefore, we investigated the mechanisms responsible for infertility in male mice after *Tmem225* deletion. We isolated and quantified sperm from the cauda epididymis and found no significant difference in sperm number between the mutant mice and the Ctrl mice (Fig. 3*A*). Subsequently, the sperm were added to HTF medium and evaluated for motility in the supernatant. Video capture of sperm movements showed that the motility of the sperm from the KO mice was significantly weaker than that of the sperm from the Ctrl mice (Supplemental Movies S1 and S2). The sperm of the Ctrl mice moved forward, while the sperm of the KO mice were much more stationary under the same conditions (Fig. 3*B*). Various indicators of sperm motility were analyzed using a computerized analysis system. The results showed that the proportion of motile sperm and progressive sperm was significantly lower in the KO mice than in the Ctrl mice (Fig. 3*C*). The average path velocity, straight-line velocity and curvilinear velocity were significantly lower in the KO mice than in the Ctrl mice (Fig. 3*D*). Acrosomal vesicle protein 1 immunofluorescence staining revealed no significant difference between the Ctrl and KO testes (Supplemental Fig. S2*A*). The acrosome reaction of the sperm was then tested, and the results showed that the rate of sperm displacement induced by progesterone was not significantly different between the KO group and the Ctrl group (Supplemental Fig. S2, *B* and *C*). In addition, we performed TEM analysis of the cauda epididymis, and the results showed that the sperm head morphology of the Ctrl and KO mice was intact (Supplemental Fig. S3, *A* and *B*). The KO mice had a 9 + 2 microtubule structure in the principal piece of sperm, which was not significantly different from that of the Ctrl group (Supplemental Fig. S3, *C* and *D*).

However, TEM revealed the presence of two tail sections in a single sperm due to tail folding (Supplemental Fig. S3, *E* and *F*). This finding suggested an abnormal tail structure in the KO sperm, which may be one of the reasons for the decreased motility. To further confirm that there was no change in sperm count in the epididymis. The caput, corpus, and cauda of the epididymal paraffin sections were stained *via* H&E staining. The nucleus was stained blue by hematoxylin, while other eosinophilic substances in the cytoplasm were stained red by eosin. H&E staining revealed that sperm were present in the caput, corpus, and cauda of the KO mouse epididymis (Fig. 3, *E–G*). Morphological analysis of the sperm was performed by Coomassie brilliant blue G250 staining. The results showed that the sperm in the caput epididymis had a standard morphology (Fig. 3*H*). Intriguingly, the sperm in the corpus and cauda epididymis had a certain degree of abnormal flagellar angulation (Fig. 3, *I* and *J*). Therefore, we divided the flagella into two types according to the degree of flagellar bending: V-shaped and hairpin. If the bending angle was 180°, the flagellum was defined as having a hairpin structure. A flagellum with a bending angle less than 180° was defined as a V-shaped structure. Counting revealed that the morphology of sperm in the caput epididymis of the TMEM225-null mice was not different from that in the caput epididymis of the Ctrl mice (Fig. 3*K*). However, in the corpus epididymis, the proportion of V-shaped KO sperm significantly increased (Fig. 3*L*). In the cauda epididymis, the proportion of V-shaped and hairpin flagella in the KO mouse sperm significantly increased (Fig. 3*M*). These results indicate that the flagella of sperm from mice lacking TMEM225 bend gradually to form a hairpin structure during movement from the caput to the cauda of the epididymis (Fig. 3*N*).

### Abnormal Flagella and Metabolism-Related Proteins in KO Sperm

**Fig. 4:**
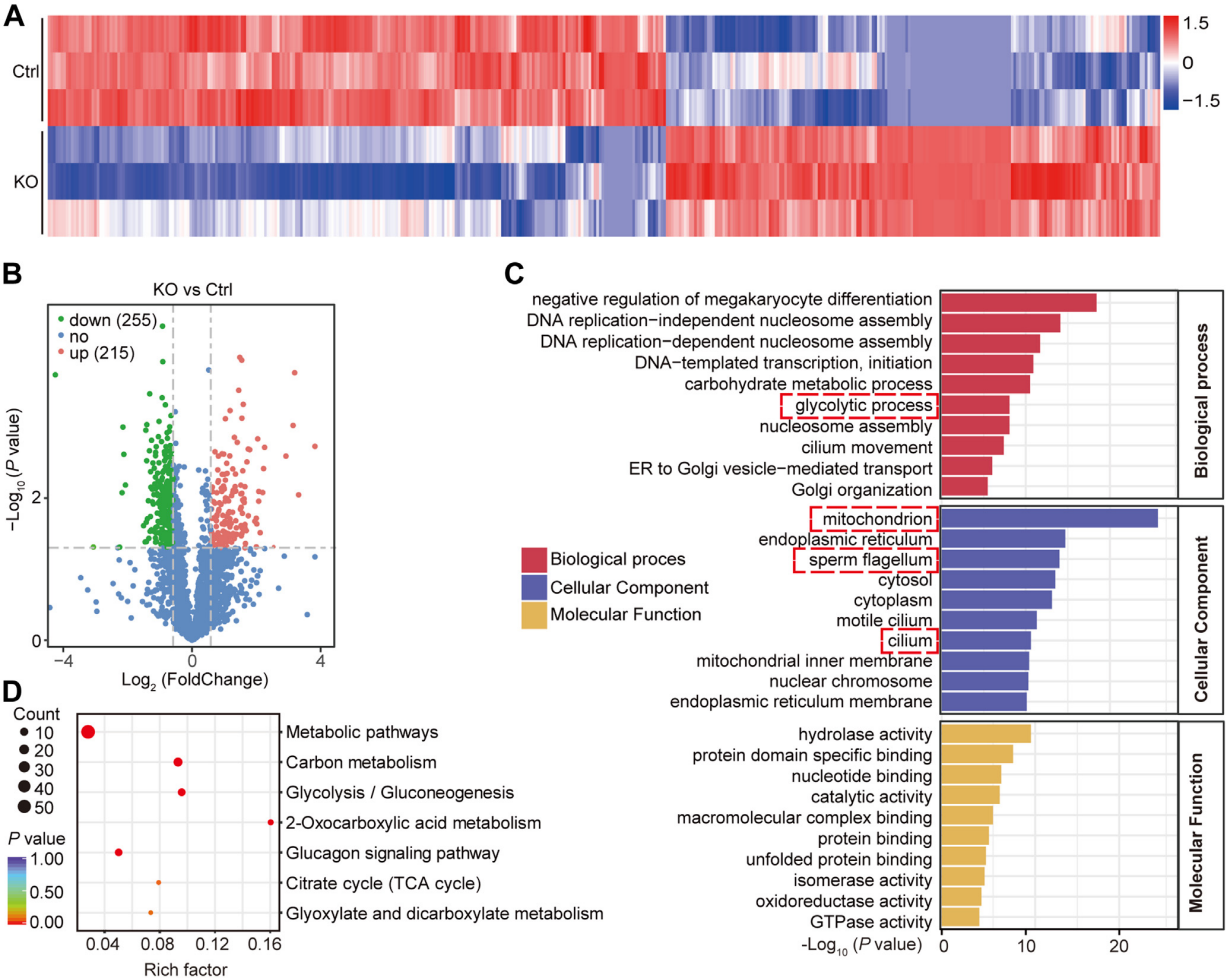
**Label-free analysis of proteomic differences in spermatozoa from TMEM225-null mice.***A*, heatmap showing 470 proteins significantly (FDR ≤ 0.05) differentially expressed between the Ctrl and KO mice. Each row of the heatmap represents the z score-transformed log_10_ values of one differentially expressed gene across all samples (*blue*, low expression; *red*, high expression). *B*, volcano plots of significantly differentially expressed proteins (FDR ≤ 0.05 and |FC| ≥ 1.5; *red*, upregulated; *green*, downregulated). *C*, GO enrichment. Each bar indicates a CC/MF/BP term, and the *abscissa* represents the number of enriched proteins. *D*, bubble plot of the KEGG pathway enrichment analysis. The above *dashed red line* indicates events associated with the ATP reduction phenotype. BP, biological process; CC, cellular component; FDR, false discovery rate; GO, Gene Ontology; KEGG, Kyoto Encyclopedia of Genes and Genomes; MF, molecular function; TMEM225, transmembrane protein 225.

To verify the molecular mechanism of TMEM225 KO-induced asthenospermia in mice, we isolated sperm from the cauda epididymis of 2-month-old mice for label-free quantitative proteome analysis. The accuracy and stability of the mass spectra and total ion chromatograms were evaluated, and all the samples met the detection standards (Supplemental Fig. S4, *A* and *B*). The mass spectrum data were highly consistent (Supplemental Fig. S4*C*). A total of 2720 proteins (with at least one unique peptide) were identified by liquid chromatography tandem mass spectrometry and considered quantified if present in at least two biological replicates (Supplemental Table S3). Perseus software (https://www./perseus/) was used to visualize the data, and Pearson correlation was used to measure the correlation. The analysis showed that the data had suitable repeatability (Supplemental Fig. S4D). The quantitative protein data were analyzed by a group *t* test, and the proteins with a *p* value≤0.05 were screened and visualized in a volcano plot (Supplemental Table S4). A heatmap of all differentially expressed proteins was generated after the Z score was transformed into the log_10_ value (Fig. 4*A*). A total of 470 differentially expressed proteins were identified; 255 were upregulated and 215 were downregulated (Fig. 4*B*). Gene Ontology (GO) enrichment analysis was performed for the differentially expressed proteins. DAVID software (david.ncifcrf.gov) was used to perform GO enrichment analysis of the screened differentially expressed proteins (Supplemental Table S5). The top 10 enriched molecular function, cellular component, and biological process GO terms were subsequently determined. The results showed that the differentially expressed proteins in the sperm of the TMEM225-null mice were enriched in glycolytic processes, mitochondria, sperm flagella, and cilia (Fig. 4*C*). Kyoto Encyclopedia of Genes and Genomes (KEGG) enrichment analysis was performed on the selected differentially expressed proteins and revealed that the sperm phenotype of the *Tmem225* deletion mice was related to metabolic pathways (Fig. 4*D*). Both GO analysis and KEGG analysis suggested that the sperm flagellum and its metabolic pathway may be key parts of the abnormalities observed in KO mice.

### Loss of TMEM225 Impaired Sperm Glycolysis and Resulted in Decreased ATP Levels

**Fig. 5:**
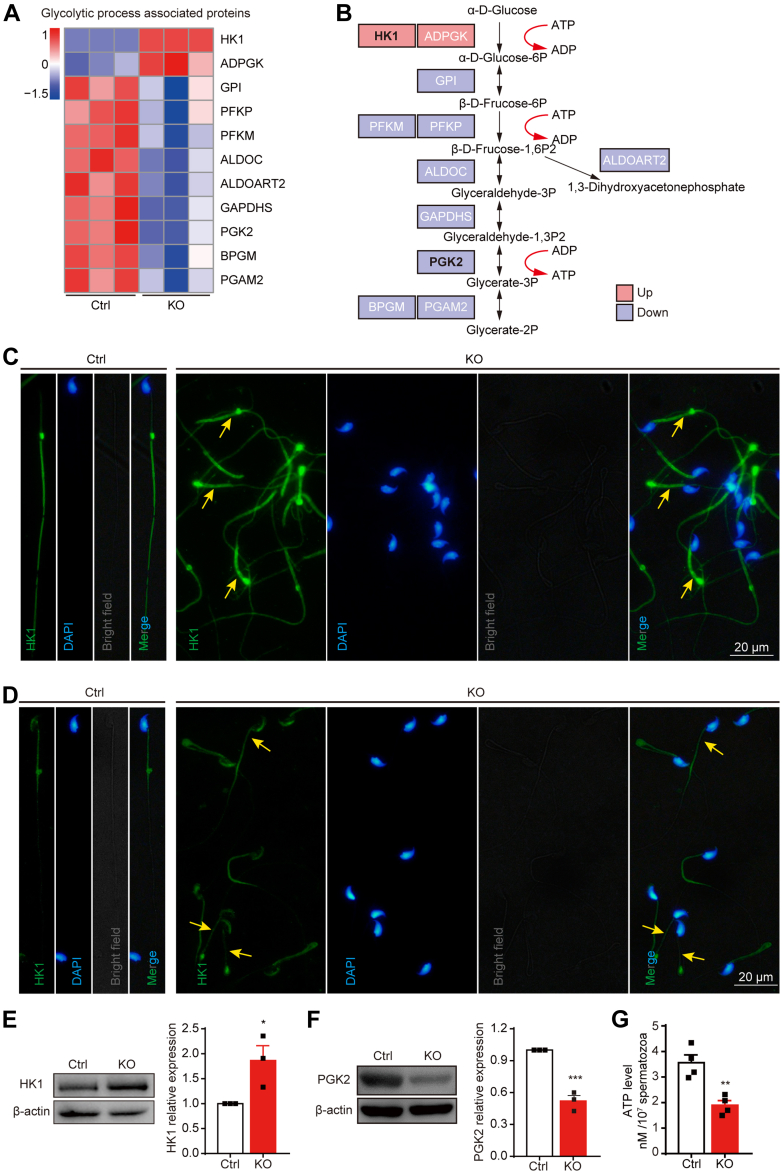
**Effects of *Tmem225* deletion on the glycolysis pathway.***A*, heatmap of GO-enriched proteins associated with the glycolytic process. *B*, diagram of the pattern of changes in the protein concentration during glycolysis. *C*, immunofluorescence was used to determine the expression and localization of HK1 in the cauda sperm of the Ctrl and KO mice. *Yellow arrows*, high expression of HK1 in sperm. *D*, immunofluorescence was used to determine the expression and localization of PGK2 in the Ctrl and KO mice. *Yellow arrows*, low expression of PGK2 in sperm. *E* and *F*, the protein expression levels of HK1 and PGK2 were analyzed *via* Western blotting and quantified *via* ImageJ. n = 3. *G*, an ATP detection kit was used to measure the relative level of ATP in the Ctrl and KO sperm. n = 4. All the data are presented as the means ± SEMs (∗*p* < 0.05; ∗∗*p* < 0.01; ∗∗∗*p* < 0.001). GO, Gene Ontology; HK1, hexokinase 1; PGK2, phosphoglycerate kinase 2; TMEM225, transmembrane protein 225.

Both GO and KEGG enrichment analyses revealed that the genes differentially expressed in response to *Tmem225* deletion were associated with multiple metabolic pathways, among which glycolysis is a crucial pathway for energy provision by spermatozoa (23). We speculated that abnormal glycolysis underlies the low sperm motility in the KO mice. To verify this hypothesis, we used a heatmap to visualize the enriched proteins. As shown by the heatmap, the levels of most of these proteins were significantly decreased (Fig. 5*A*). The differentially expressed proteins were mapped according to the glycolysis pathway (Fig. 5*B*). According to the atlas, HK1, which catalyzes the first step of glycolysis, and phosphoglycerate kinase 2 (PGK2), which catalyzes the first step of ATP production, were selected for verification. Immunofluorescence analysis was used to demonstrate the localization of HK1 and PGK2. We found that HK1 was continuously localized to the flagella of sperm from the Ctrl mice, and the positive signal in the principal piece was stronger than that in the midpiece. However, in the KO sperm, the positive signal in the midpiece was significantly stronger than that in the principal piece (Fig. 5*C*). Immunofluorescence analysis of PGK2 expression in sperm revealed that PGK2 in the flagella of the KO mice was discontinuously positioned in the midpiece (Fig. 5*D*). Western blotting analysis revealed that the relative protein expression level of HK1 increased, while the protein expression level of PGK2 decreased (Fig. 5, *E* and *F*). Since glycolysis is an essential pathway for ATP production in sperm and since the expression of two key enzymes involved in glycolysis is abnormal, we hypothesized that ATP levels in the sperm of the KO mice would be abnormal. We next examined the ATP level in sperm in the cauda epididymis and found that the ATP level in the KO mice was significantly lower than that in the Ctrl mice (Fig. 5*G*). Specifically, abnormal levels of enzymes related to glycolysis affect sperm ATP production, which is an important cause of asthenospermia.

### Disruption of *Tmem225* Impaired Mitochondrial Function

**Fig. 6:**
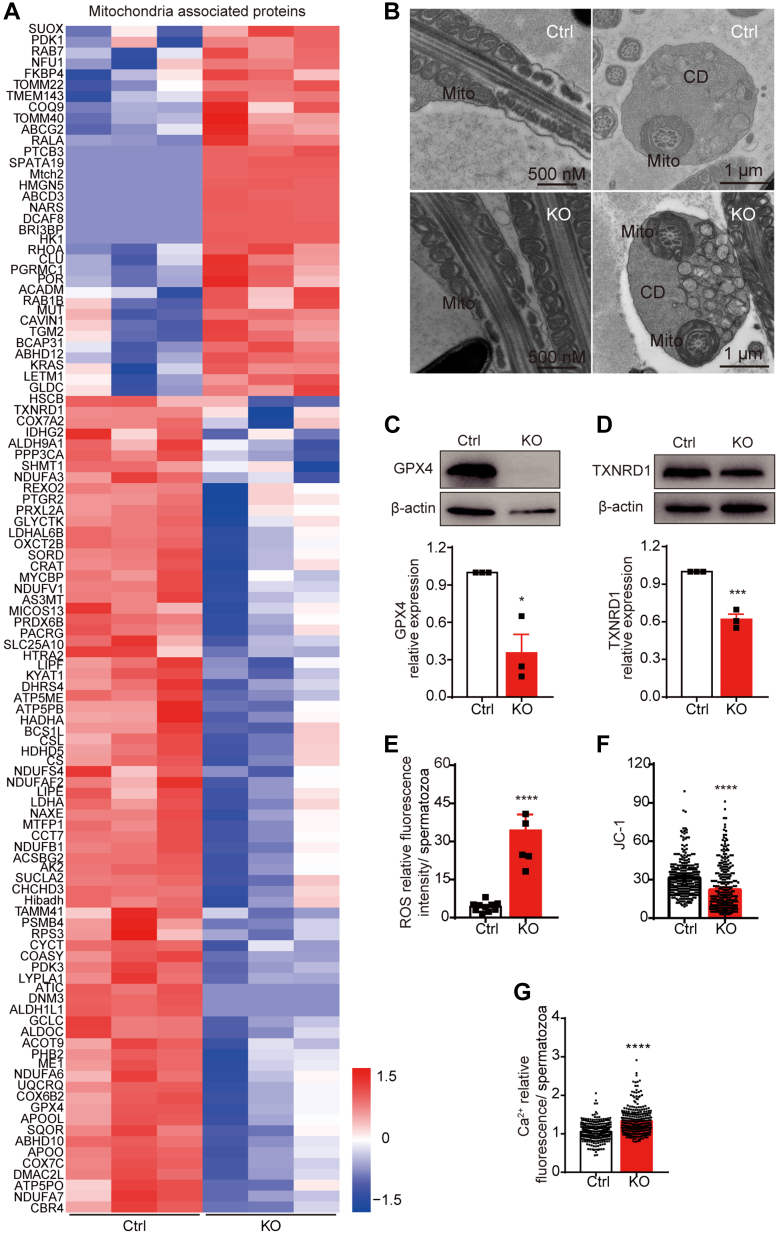
**Effects of TMEM225 deficiency on the mitochondria.***A*, GO enrichment analysis was used to construct a heatmap of the differentially expressed proteins related to mitochondria. *B*, mitochondrial ultrastructure of sperm in the cauda epididymis. Mito, mitochondria; CD, cytoplasmic droplets. *C* and *D*, the protein expression levels of GPX4 and TXNRD1 were analyzed *via* Western blotting and quantified *via* ImageJ. n = 3. *E*, ROS levels in cauda epididymis spermatozoa. Ctrl: n = 10, KO: n = 5. *F*, A JC-1 kit was used to determine the relative MMP. A total of 320 spermatozoa were assessed in three biological replicates of the Ctrl group. A total of 310 spermatozoa were assessed in three biological replicates of the KO group. *G*, the intracellular calcium level was determined by Fluo-4 labeling. A total of 301 spermatozoa were assessed in three biological replicates of the Ctrl group. A total of 300 spermatozoa were assessed in three biological replicates of the KO group. All the data are presented as the mean ± SEM (∗∗∗∗*p* < 0.0001). GO, Gene Ontology; MMP, mitochondrial membrane potential; ROS, reactive oxygen species; TMEM225, transmembrane protein 225; TXNRD1, thioredoxin reductase 1.

Previous studies have shown that a decrease in ATP levels may be related to abnormal mitochondrial membrane structure and may also be caused by abnormal mitochondrial function (24, 25). In our study, 216 genes involved in mitochondria-related pathways were found to be abnormally expressed in KO mouse sperm (Fig. 6*A*). Therefore, we first used TEM to assess mitochondrial morphology, and the results showed that the membrane structure and morphology of mitochondria in KO and Ctrl sperm were consistent (Fig. 6*B*). However, the protein levels of phospholipid hydroperoxide glutathione peroxidase (GPX4) and thioredoxin reductase 1 in the cauda epididymal sperm from the KO mice were significantly lower than those in the cauda epididymal sperm from the Ctrl mice, as verified by Western blotting (Fig. 6, *C* and *D*). Consequently, we examined the level of ROS in sperm and found a significant increase in the sperm from the KO mice (Fig. 6*E*). The mitochondrial membrane potential (MMP) may decrease due to increased ROS levels. We then examined the relative MMP using a JC-1 kit. The results showed that the MMP was significantly lower in the KO mice than in the Ctrl mice (Fig. 6*F*). The Fluo-4 assay also showed a significant increase in the Ca^2^⁺ concentration in the sperm of the KO mice (Fig. 6*G*). These results confirmed our hypothesis that sperm mitochondrial function is abnormal in TMEM225-null mice.

### Deletion of *Tmem225* Resulted in the Bending of the Sperm Flagellum

**Fig. 7:**
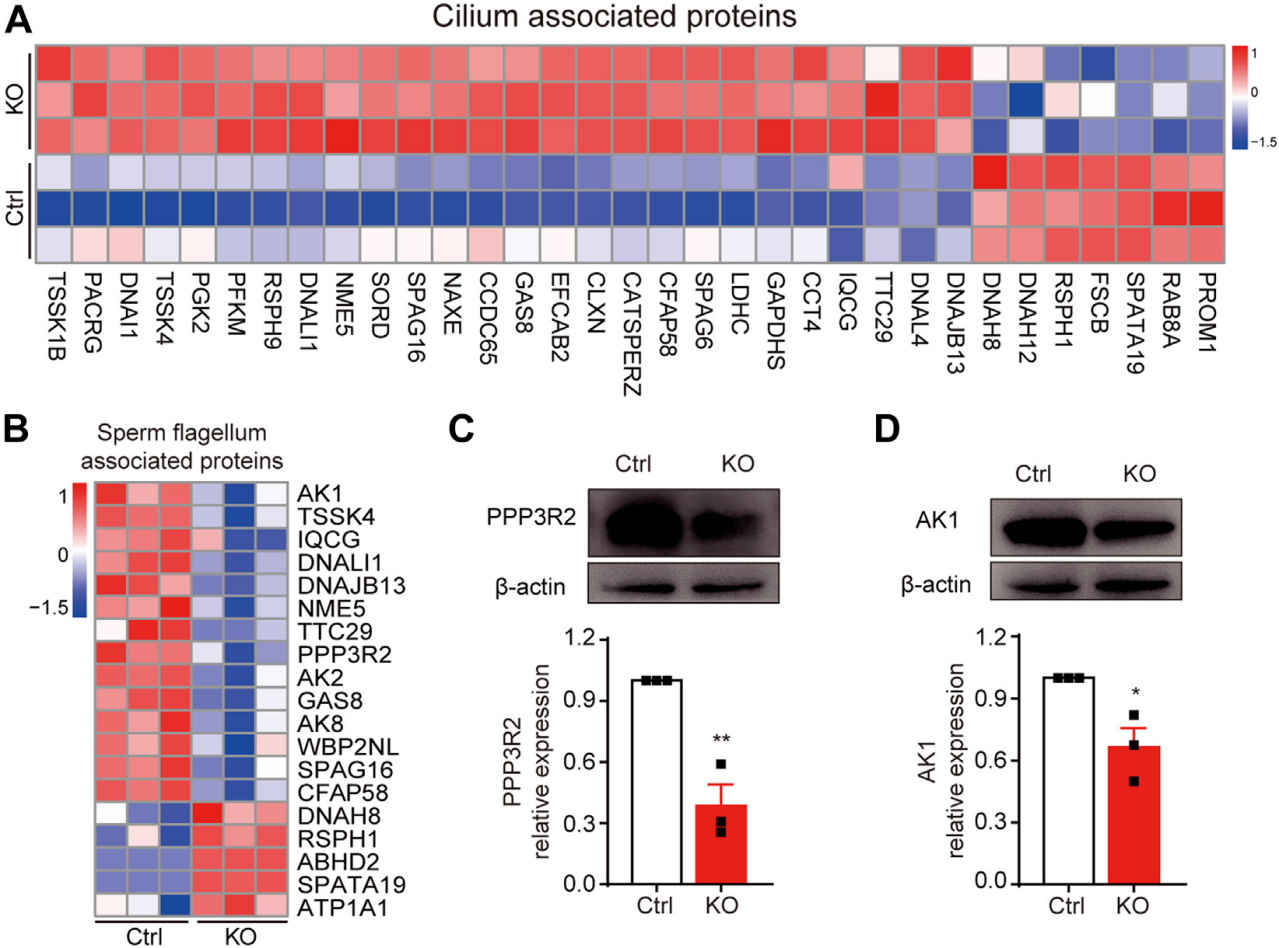
**Effects of TMEM225 deficiency on the cilium.***A*, GO enrichment analysis was used to construct a heatmap of the differentially expressed proteins related to the cilium. *B*, GO enrichment analysis revealed a heatmap of differentially expressed proteins related to the sperm flagellum. *C* and *D*, the protein expression levels of PPP3R2 and AK1 were analyzed *via* Western blotting and quantified *via* ImageJ. n = 3. All the data are presented as the means ± SEMs (∗*p* < 0.05; ∗∗*p* < 0.01). AK1, adenylate kinase 1; GO, Gene Ontology; PPP3R2, protein phosphatase 3 regulatory subunit B beta isoform; TMEM225, transmembrane protein 225.

Many previous studies have shown that abnormalities in the sperm tail are also essential factors for decreased sperm motility (26, 27). Our GO enrichment analysis revealed that many components were associated with the sperm tail. The structural components involved in sperm bending include cilia and sperm flagella (Fig. 7, *A* and *B*). Among the differentially expressed proteins enriched in the sperm flagellum, protein phosphatase 3 regulatory subunit B beta isoform (PPP3R2) and adenylate kinase 1 (AK1) are related to sperm flagellum bending (26, 28). Therefore, a Western blotting assay was used to determine the expression levels of these two proteins in the sperm of the cauda epididymis. The results showed that the protein expression levels of PPP3R2 and AK1 in the sperm from the KO mice were significantly lower than those in the sperm from the Ctrl mice (Fig. 7, *C* and *D*). These findings suggested that a lack of sperm flagellum-related proteins may lead to folding of the sperm flagellum in KO mice.

## Discussion

In clinical diagnosis, oligospermia, asthenospermia, and teratozoospermia are the main causes of male infertility. Asthenospermia is characterized by a severe decrease in sperm motility or immobility, which is often accompanied by structural abnormalities of the flagella (29). Up to 80% of male infertility cases are due to asthenospermia, which varies in severity (30). Various genetic abnormalities are associated with asthenospermia in humans, but the underlying molecular mechanisms remain unclear due to the lack of *in vitro* models. In this study, a *Tmem225* KO mouse model was constructed to determine the essential role of TMEM225 in male fertility. Our study revealed that the absence of TMEM225, a membrane protein expressed solely during spermiogenesis, can lead to the development of classic asthenospermia in mice. Specifically, TMEM225-deficient mice were infertile, and sperm collected from the cauda epididymis exhibited significantly reduced viability, varying degrees of tail folding, and high ROS levels.

Our results showed that TMEM225 was expressed in elongated spermatids. This finding is consistent with those of previous studies (20, 21). These results suggested that TMEM225 plays a crucial role in spermiogenesis. Interestingly, morphological analysis of testes and immunofluorescence staining of sperm from TMEM225-null mice revealed no damage to spermiogenesis. The morphology of the sperm head, mitochondrial arrangement, and 9 + 2 microtubule structure were typical. After sperm enter the epididymis, the bending angle and abnormal proportion of sperm cilia increase significantly as the sperm moves from the caput epididymis to the cauda epididymis. By using label-free proteomics analysis of sperm in the cauda epididymis, we found significant differences in the proteome between the KO and Ctrl mice. The differentially expressed proteins were shown to be associated with cilium and sperm flagellum processes through GO enrichment analysis. Deletion of some of these proteins has been reported to be associated with sperm cilium bending. Previous studies have shown that AK1 and AK2 are accessory structures of sperm flagella (31). Moreover, outer dense fiber 4 deficiency was reported to lead to the bending and low motility of sperm flagella by reducing AK1 and AK2 protein levels (26). A lack of PPP3R2 resulted in decreased motility and eventual infertility in male mice (28). One cause of the reduced PPP3R2-null sperm motility is the limited sperm tail oscillations after folding (27). These results suggest that the cilium folding phenotype caused by *Tmem225* deletion may be a result of abnormal levels of these proteins.

In addition, the GO analysis of the proteomics-enriched proteins revealed their relationship with glycolysis. Glycolysis is a critical pathway in which sperm produce ATP and provide energy for movement. Previous studies have shown that the locations of glycolysis-related proteins in sperm substantially overlap with those in the sperm flagellum (5, 32). The results of immunofluorescence staining for HK1 and PGK2 were also consistent with these findings. HK1, the enzyme needed for the first step of glycolysis, functions by consuming ATP. PGK2, the PGK isoenzyme in sperm, catalyzes the first ATP production step in the glycolytic pathway. The absence of this enzyme leads to decreased sperm motility and male fertility in mice (33). Our results showed that HK1 protein levels increased and PGK2 protein levels decreased, which resulted in the decrease in sperm ATP levels. Furthermore, the expression of GAPDHS, which was downregulated in sperm with *Tmem225* deletion, resulted in reduced sperm motility and infertility in mice (34). According to our results, *Tmem225* deletion leads to decreased activity by affecting glycolytic pathway protein levels.

We also found that mitochondria-related proteins were enriched among the differentially expressed proteins, and GPX4 and thioredoxin reductase 1 are associated with inhibiting ROS production and alleviating the oxidative stress response (35, 36). GPX4 deficiency also led to infertility in male mice (37, 38). A reduction in the levels of these two proteins led to high ROS levels in the sperm of *Tmem225* KO mice. Many patients with infertility also have high ROS levels in their sperm (39). Moreover, although the loss of TMEM225 did not damage mitochondrial structure, it suppressed mitochondrial function. Mitochondrial dysfunction may also contribute to the reduction in ATP levels.

In conclusion, our results demonstrate that *Tmem225* deletion does not affect spermatogenesis but causes asthenospermia and leads to infertility in male mice through changes in the proteins in epididymal sperm. This study provides new insights for the potential development of a TMEM225-based reversible nonhormonal contraceptive.

## Data Availability

The mass spectrometry proteomics data have been deposited in the ProteomeXchange Consortium (http://proteomecentral.proteomexchange.org) *via* the iProX partner repository with the dataset identifier PXD046484. URL: https://www.iprox.cn/page/SSV024.html;url=16987192700135da1, password: Fzvg. Annotated spectral files was uploaded to the MS viewer. The search key for the saved data set is mf4hjpkave.

## Supplemental data

This article contains supplemental data.

## Conflict of interest

The authors declare no competing interests.

## Supplementary Data

Supplemental Table S5

KO cauda sperm.avi

Het cauda sperm.avi
