# Supplementary material for: TMEM225 Is Essential for Sperm Maturation and Male Fertility by Modifying Protein Distribution of Sperm in Mice
Source: Mol Cell Proteomics. 2024 Jan 20;23(2):100720. doi: 10.1016/j.mcpro.2024.100720 (PMC10875271; doi:10.1016/j.mcpro.2024.100720)
Supplement: Supplemental Data [file mmc1.docx]

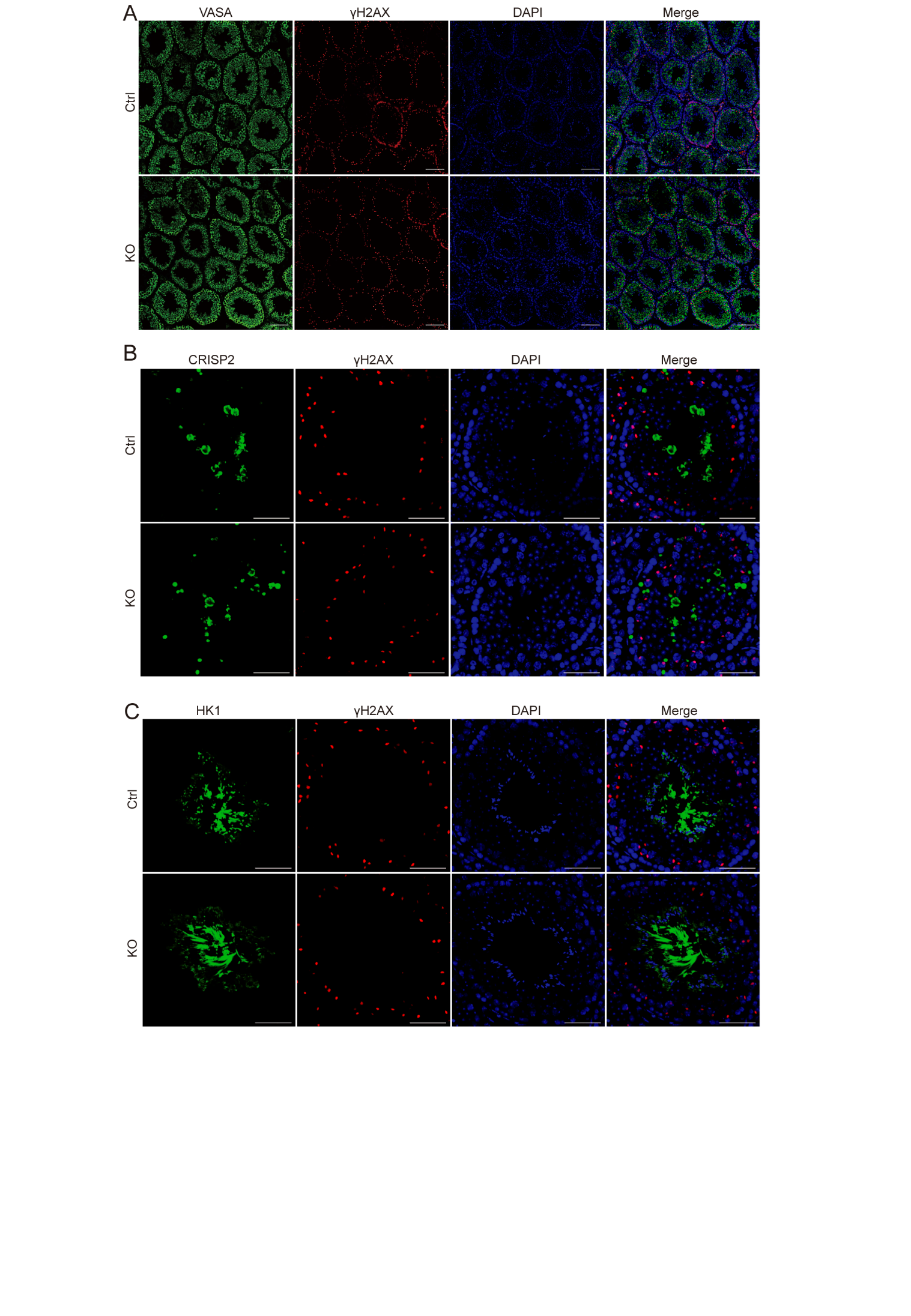


F_IG_. S1. **Immunofluorescence staining of markers associated with spermiogenesis**. *A* Co-immunofluorescence staining of the germ cell marker VASA and the DNA double-strand break marker γH2AX. Bar=200 μm. *B* Co-immunofluorescence staining of the late spermiogenesis markers CRISP2 and γH2AX. Bar=40 μm. *C* Co-immunofluorescence staining of the late spermiogenesis markers HK1 and γH2AX. Bar=40 μm.


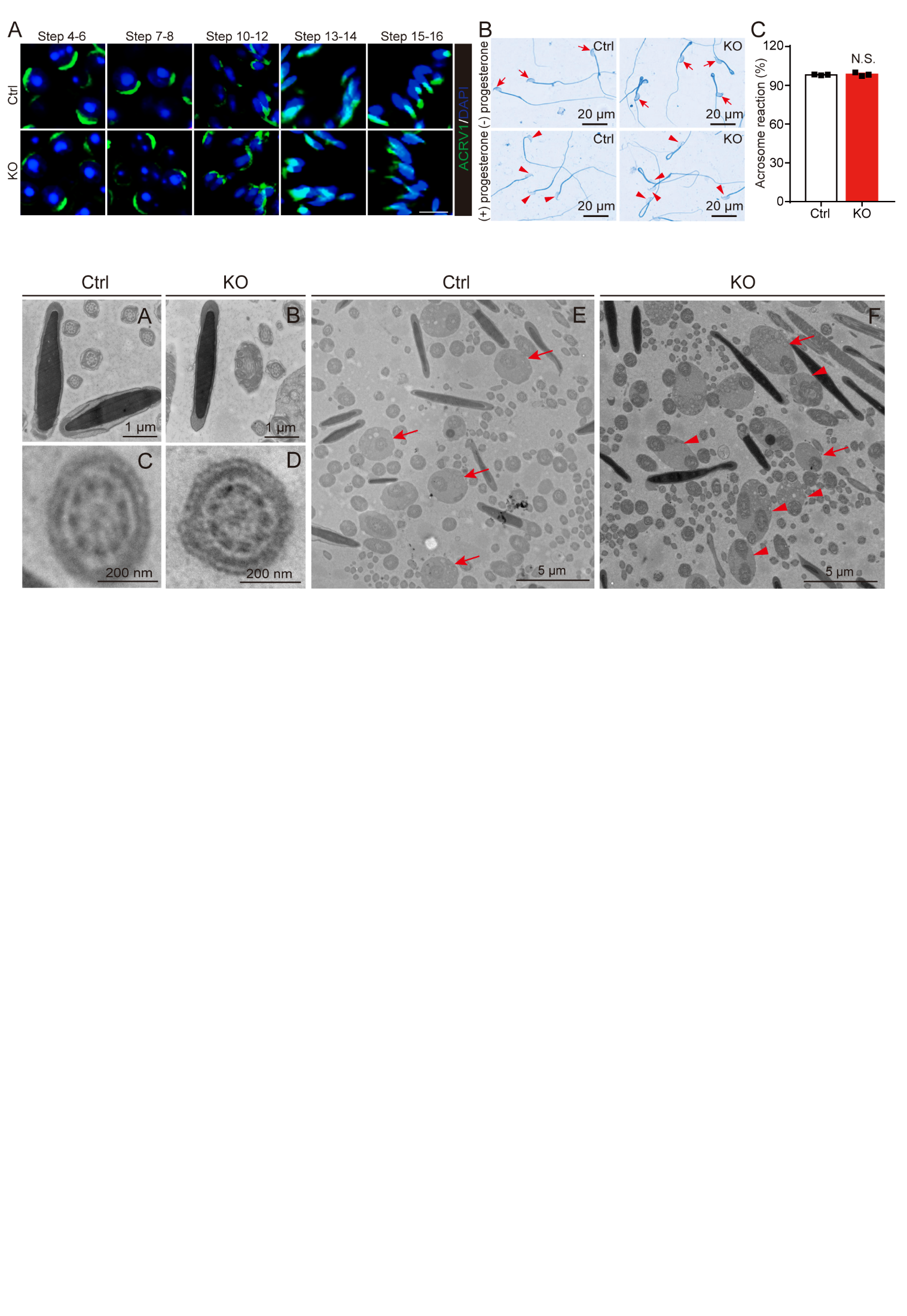


F_IG_. S2. **Acrosome formation and reaction.** *A* Immunofluorescence staining of the acrosome marker ACRV1. Bar=10 μm. *B* The acrosome reaction of sperm was induced by progesterone. *C* The proportion of acrosome reactions and the significance of differences were analyzed. Red arrows, morphology before acrosome reaction; red arrowheads, morphology after acrosome reaction. Data are presented as the mean ± SEM (N.S., no significant difference).


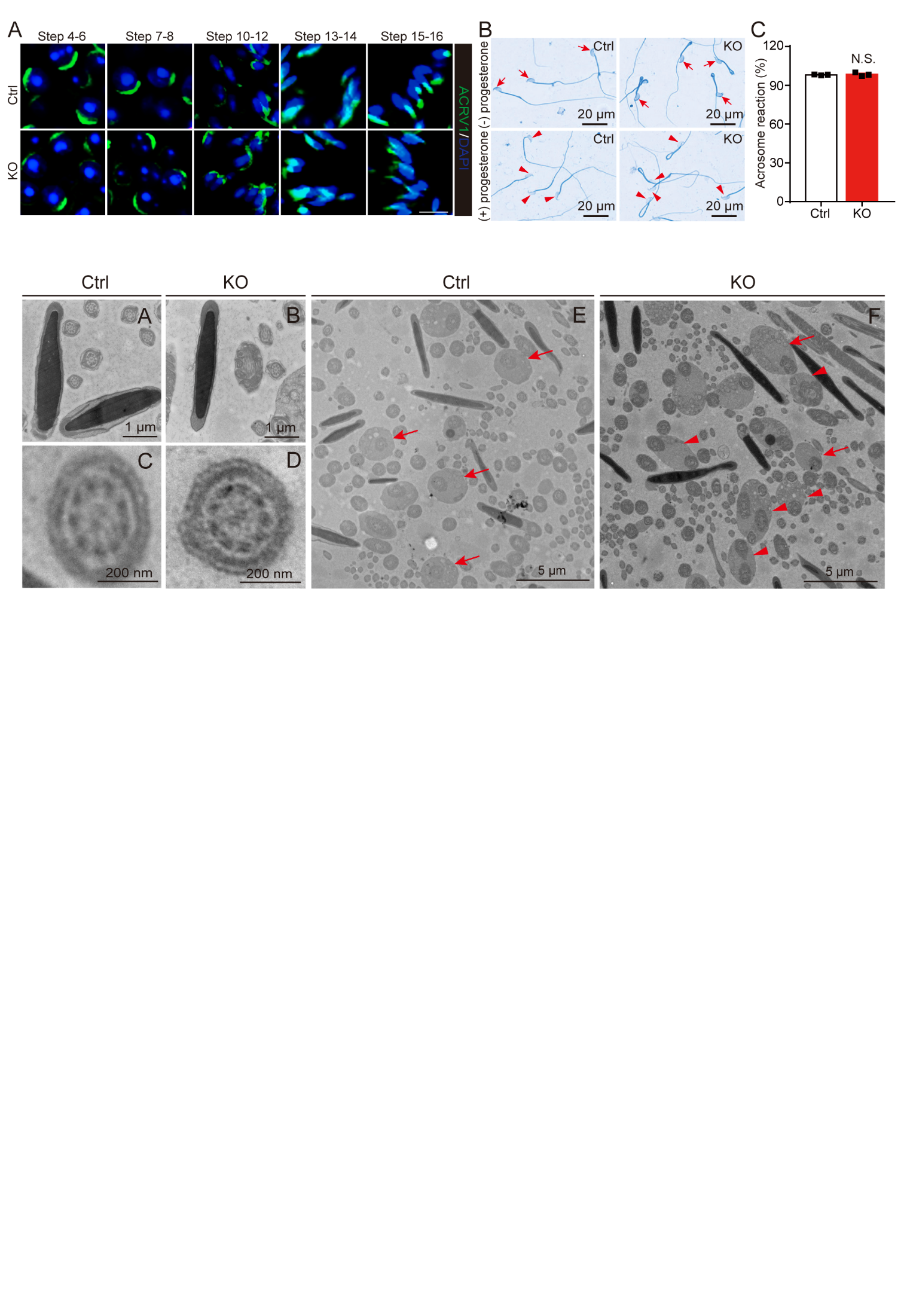


F_IG_. S3. **The ultrastructure of sperm was analyzed by TEM.** *A-B* TEM images of Ctrl and KO sperm heads. *C-D* TEM images of Ctrl and KO sperm principal pieces. *E-F* TEM images showed sperm in the cauda epididymis. Red arrows, normal CD with tail; red arrowheads, CD with hairpin tail.


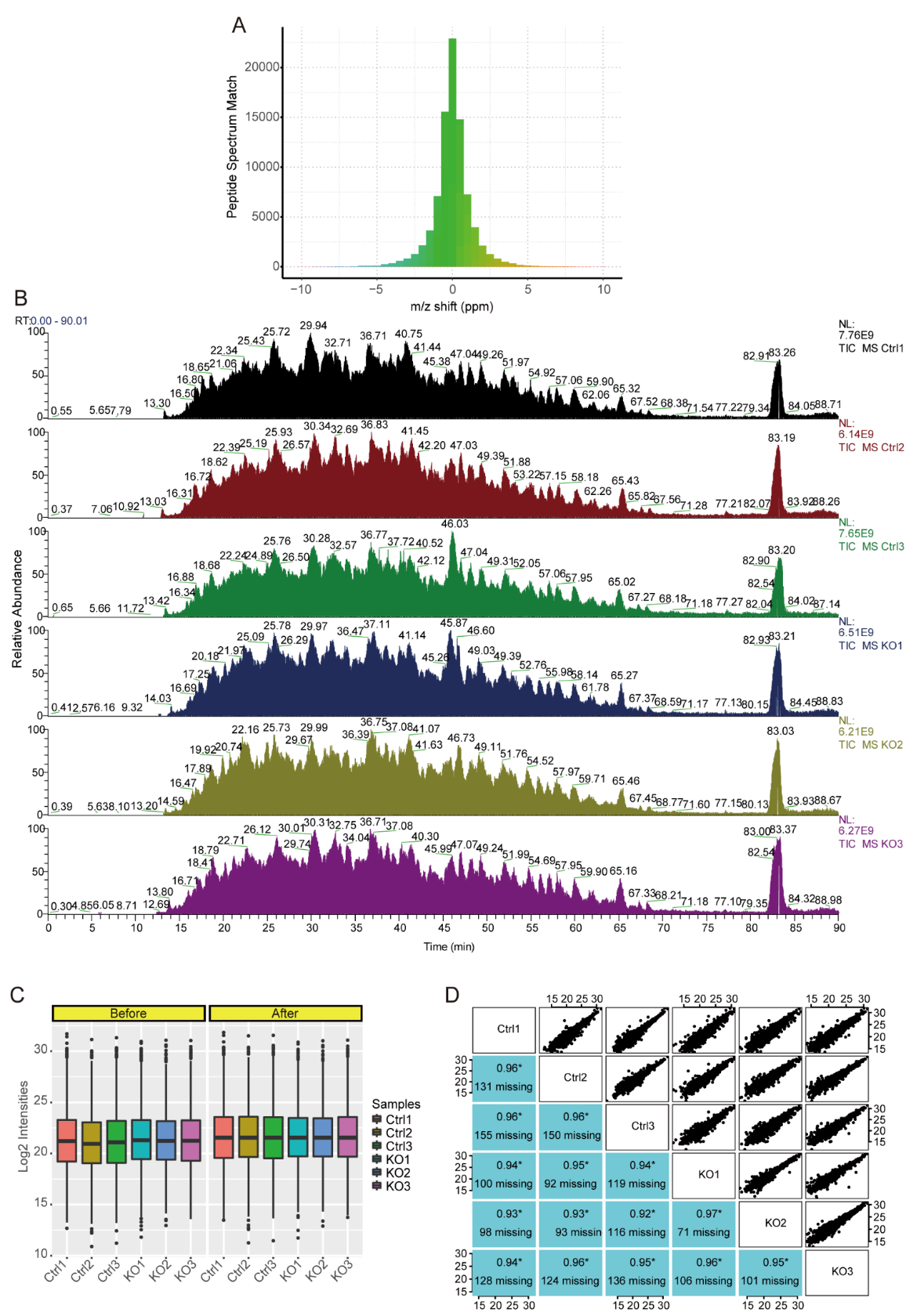
F_IG_. S4. **Caudal sperm proteomic analysis.** *A* Evaluation of the stability of mass spectrometry accuracy. The horizontal axis is the mass error (ppm), and the vertical axis is the peptide spectrum match. *B* Total ion chromatogram. The abscissa is the retention time, and the ordinate is the signal intensity. *C* The experimental treatments were controlled in parallel. The abscissa is the retention time, and the ordinate is the signal intensity. *D* Correlation analysis. Each point in the scatter plot represents a protein, the horizontal and vertical coordinates are the log_2_ pairs of the relative quantitative values of parallel sample proteins, and the values in the figure are the values of the phase relationship between corresponding samples.


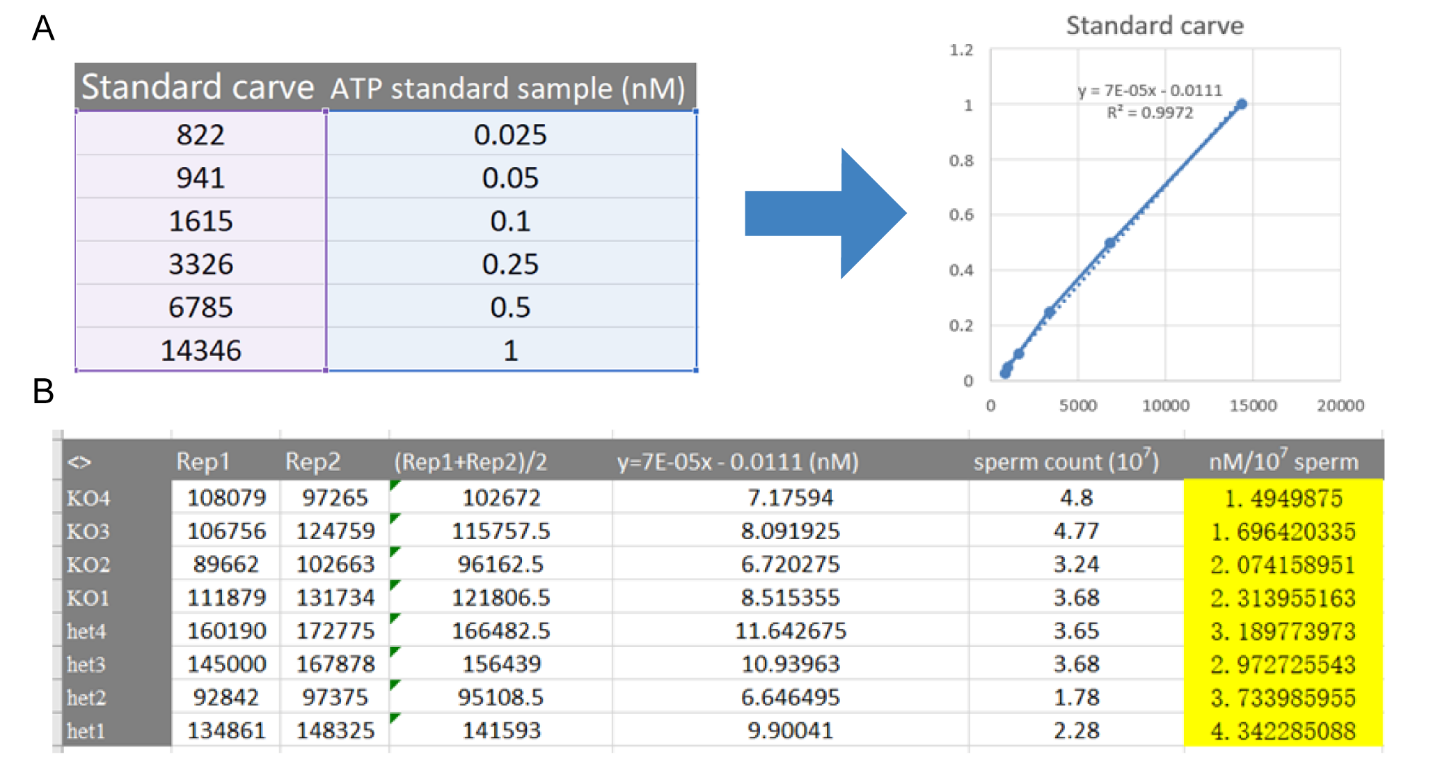


F_IG_. S5. **Calculation of ATP levels in Ctrl and KO sperm.** *A* A standard curve was drawn by ATP standard samples. *B* ATP content per sperm was calculated from the standard curve and sperm count.


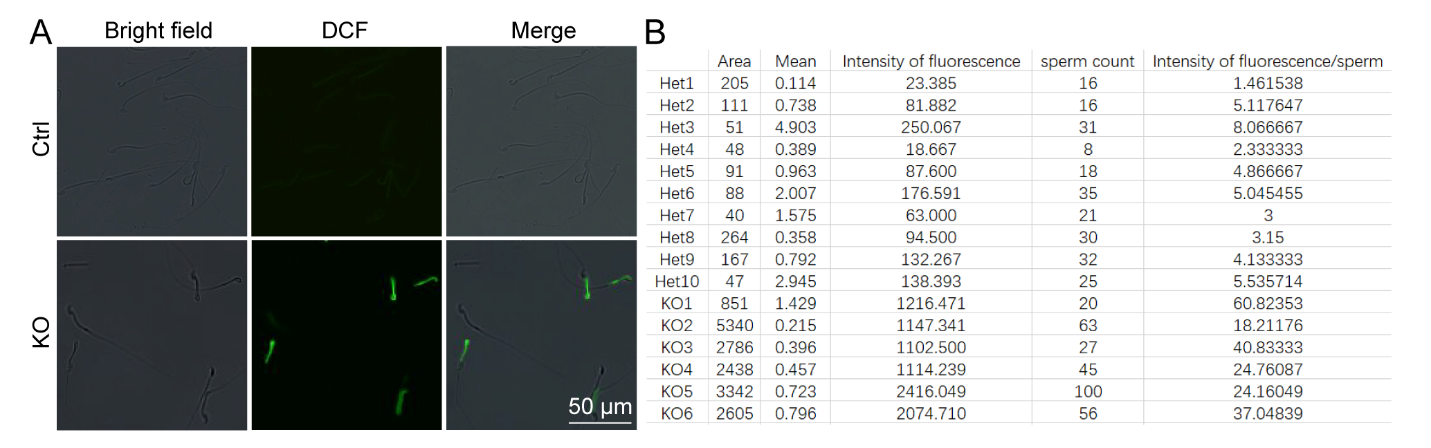


F_IG_. S6. **Analysis of ROS level.** *A* DCF fluorescence intensity was used to detect ROS levels in Ctrl and KO sperm. *B* DCF fluorescence intensity was analyzed by ImageJ.

Table S1. Primers for *Tmem225* genotyping

| F | CCTCATGAAAGTTTGACCAAGACAG |
| --- | --- |
| R1 | GATTGGTTACATACAGCTAATTCCATAG |
| R2 | GCAGAATAGAACCCACAAGCCC |

Table S2. Antibodies used in this study

| Name | Cat.NO. | Source | Company | Dilution |
| --- | --- | --- | --- | --- |
| GAPDH | 60004-1-Ig | Mouse monoclonal | Proteintech | WB, 1: 10000 |
| ACTB | 81115-1-RR | Rabbit monoclonal | Proteintech | WB, 1: 20000 |
| TXNRD1 | 11117-1-AP | Rabbit polyclonal | Proteintech | WB, 1: 6000 |
| AK1 | 14978-1-AP | Rabbit polyclonal | Proteintech | WB, 1: 2000 |
| PPP3R2 | 14005-1-AP | Rabbit polyclonal | Proteintech | WB, 1: 2000 |
| GPX4 | ab125066 | Rabbit polyclonal | abcam | WB, 1: 1000 |
| CRISP2 | 19066-1-AP | Rabbit polyclonal | Proteintech | IF, 1:200 |
| VASA | ab13840 | Rabbit polyclonal | abcam | IF, 1:300 |
| ACRV1 | 14040-1-AP | Rabbit polyclonal | Proteintech | IF, 1:100;  WB, 1: 2000 |
| HK1 | ab150423 | Rabbit polyclonal | abcam | IF, 1:100;  WB, 1: 2000 |
| PGK2 | 13686-1-AP | Rabbit polyclonal | Proteintech | IF, 1:100;  WB, 1: 2000 |
| γH2AX  (AlexaFluor® 555) | 05-636-AF555 | Mouse monoclonal | Millipore | IF, 1:800 |
| GAM-HRP | 7076P2 | Goat polyclonal | CST | WB, 1: 2000 |
| GAR-HRP | 7074P2 | Goat polyclonal | CST | WB, 1: 2000 |
| GAR-488 | A11034 | Goat polyclonal | Life Technologies | IF, 1: 500 |
